# Areca Users in Combination with Tobacco and Alcohol Use Are Associated with Younger Age of Diagnosed Esophageal Cancer in Taiwanese Men

**DOI:** 10.1371/journal.pone.0025347

**Published:** 2011-10-19

**Authors:** Ming-Yen Lin, Mei-Chin Chen, I-Chen Wu, Deng-Chyang Wu, Yu-Jen Cheng, Chun-Chieh Wu, Chee-Yin Chai, Jang-Ming Lee, Ming-Tsang Wu

**Affiliations:** 1 Graduate Institute of Occupational Safety and Health, Kaohsiung Medical University, Kaohsiung, Taiwan; 2 Department of Family Medicine, Kaohsiung Medical University Hospital, Kaohsiung, Taiwan; 3 Division of Gastroenterology, Department of Internal Medicine, Kaohsiung Medical University Hospital, Kaohsiung, Taiwan; 4 Graduate Institute of Medicine, College of Medicine, Kaohsiung Medical University, Kaohsiung, Taiwan; 5 Divsion of Thoracic Surgery, E-Da Hospital, I-Shou University, Kaohsiung, Taiwan; 6 Department of Pathology, Kaohsiung Medical University Hospital, Kaohsiung, Taiwan; 7 Department of Surgery, National Taiwan University Hospital, Taipei, Taiwan; 8 Center of Environmental and Occupational Medicine, Kaohsiung Municipal Hsiao-Kang Hospital, Kaohsiung, Taiwan; Genentech Inc., United States of America

## Abstract

**Background:**

Whether the habitual use of substances (tobacco, alcohol, or areca nut (seed of the Areca palm)) can affect the age of esophageal squamous cell carcinoma (ESCC) presentation has rarely been examined.

**Methods:**

The study subjects were those who were males and the first time to be diagnosed as ESCC (ICD-9 150) and who visited any of three medical centers in Taiwan between 2000 and 2009. A standardized questionnaire was used to collect substance uses and other variables.

**Results:**

Mean age (±SD) at presentation of ESCC was 59.2 (±11.3) years in a total of 668 cases. After adjusting for other covariates, alcohol drinkers were 3.58 years younger to have ESCC than non-drinkers (*p* = 0.002). A similar result was found among areca chewers, who were 6.34 years younger to have ESCC than non-chewers (*p*<0.0001), but not among cigarette smokers (*p* = 0.10). When compared to the group using 0–1 substances, subjects using both cigarettes and alcohol were nearly 3 years younger to contract ESCC. Furthermore, those who use areca plus another substance were 7–8 years younger. Subjects using all three substances had the greatest age difference, 9.20 years younger (*p*<0.0001), compared to the comparison group.

**Conclusion:**

Our findings suggest that habitually consuming tobacco, alcohol, and areca nut can influence the age-onset of ESCC. Since the development of ESCC is insidious and life-threatening, our observation is worthy to be reconfirmed in the large-scale and long-term follow-up prospective cohort studies to recommend the screening strategy of this disease.

## Introduction

Esophageal cancer is the 6th leading cause of cancer death worldwide, estimated to be responsible for 562,000 deaths in 2008 [1]. In Taiwan, the annual incidence rate of esophageal cancer in men has increased at least 2-fold in the recent decade (from 5.7/10^5^ in 1995 to 12.1/10^5^ in 2007 (Figure S1) [2], [3]. Given the insidious nature of this neoplasm, more than 50 percent of patients have either unresectable tumors and/or metastases at presentation [4]. The overall 5-year survival rate for esophageal cancer is less than 15% [5]. However, with complete surgical removal of the tumor, the 5-year survival rate exceeds 95 percent for stage 0 disease, and is 50 to 80 percent for stage I of the disease [5].

Endoscopy assisted with mucosal iodine staining or narrow-band imaging has increased the detection rate of early esophageal cancers confined to the mucosa [6], [7], [8]. Because the majority of esophageal cancer in Taiwan is esophageal squamous cell carcinoma (ESCC) and early ESCC can be treated by endoscopic mucosal resection with a 5-year survival rate of 95–100% [9], [10], [11], [12], implementing the strategy of screening high-risk subjects can greatly improve the survival rate of this cancer.

Alcohol and tobacco use are well-recognized risk factors of ESCC around the world [13], [14], [15], [16]. Chewing of areca nut, which is the seed of the Areca palm (Areca catechu), is a prevalent habit in Taiwan. Two most common types of areca chewing are noted in Taiwan: raw betel fruit and lime with Piper betle inflorescence or folded in betel leaf. It is estimated that nearly 2,800,000 people use areca is Taiwan, more men than women (9.8% *vs.* 1.6%), with the life-time prevalence as high as 15% [17]. Around the world there were six hundred million people chewing areca nut, especially in India and Southeast Asia [18]. A series of recent studies, including ours, have shown that areca chewing is also a risk factor for ESCC [12], [19], [20], [21], [22]. In a case-control study of 513 ESCC and 818 gender- and age-matched controls, those using any two of alcohol, tobacco, or areca can contract at least a 4-fold risk of ESCC [12]. This risk increased to 41-fold for users of all three substances. The current American Cancer Society guidelines for cancer screening include cancers of the breast, cervix, colorectum, endometrium, lung, prostate, and skin, but not esophagus [23]. Two major histological types of esophageal cancer in the worldwide are esophageal adenocarcinoma and ESCC. Since Barrett's esophagus is the major risk of esophageal adenocarcinoma, screening of patients with Barrett's esophagus without epithelial dysplasia has been recommended to be performed by endoscopy every 3–5 years [24]. In contrast to esophageal adenocarcinoma, a number of risk factors for ESCC have also been recognized, but guidelines for screening of ESCC in high-risk groups are still lacking. In addition, the proper initial age and interval for screening of ESCC are yet to be defined. Thus, in this study, we analyzed the age of patients with ESCC in Taiwanese men at initial diagnosis and the influence of alcohol drinking, tobacco smoking and areca nut chewing on the age of initial presentation.

## Materials and Methods

### Study Subjects

In Taiwan, between the years 2000 and 2009, a multicenter cancer patient recruitment for a molecular epidemiologic investigation was conducted in three medical centers, National Taiwan University Hospital (NTUH) in Taipei and Kaohsiung Medical University Hospital (KMUH) and Kaohsiung Veterans General Hospital (KVGH) in Kaohsiung. The study subjects were those who were the first time to be diagnosed as ESCC (ICD-9 150) and visited these three medical centers for help [12], [19]. In order for newly diagnosed ESCC cases to be both identified and enrolled for this study as soon as the respective histopathological materials were confirmed, a review network for quick case recognition and verification was established at the Departments of Chest Surgery and Gastroenterology in these medical centers [12]. According to our previous studies [12], [19], the participated rate was 71.5% in NTUH and ∼95% in both KMUH and KVGH. Since esophageal cancer in Taiwanese women only accounted for 10% of total and their etiologies may be different from those in Taiwanese men, this study focused on the analysis of men.

Clinical and pathological features were reviewed and evaluated by independent pathologists according to the TNM staging system of the American Joint Committee on Cancer [25]. The ethics review boards at Kaohsing Medical University Hospital reviewed and approved this investigation. Written consents were obtained from all participants.

### Questionnaire

A standardized questionnaire was used to collect comprehensive information of demographic characteristics and substance use via an in-person interview with participants within 1 week of cancer diagnosis. The average length of interviews was approximately 30 min. The ethics review boards at the study hospitals reviewed and approved this investigation. Alcohol drinkers, tobacco smokers and areca chewers were defined, respectively, as subjects who had consumed any alcoholic beverage ≥1 times per week, those who had smoked ≥10 tobacco cigarettes per week and those who had chewed ≥1 areca-nut (measured as quid) per day for at least 6 months. The age at which a substance use started, type of substances, daily consumption amount and duration of such use, were documented for each participant [19]. One drink of alcohol consumption was defined as ∼14 gram of alcohol [26]. In addition, other information such as educational levels (<high school, high school, and >high school), study hospitals (NTUH, KMUH, and KVGH), and clinical stages (Stage I–IV) were also collected.

### Validation of Substance Uses

Previously, we used different biomarkers in different specimens to verify information about tobacco, alcohol, and areca from questionnaires [12], [27]. To verify the smoking status reported in the questionnaire, we measured cotinine and creatinine levels in the one-spot urine specimens of 22 active smokers and 74 non-smokers from one previous community study [27]. Urinary cotinine and creatinine were measured by liquid chromatography tandem mass spectrometry (LC/MS/MS) equipped with a triple-quadruple mass spectrometer and TurboIonSpray™ (API 3000™, Applied Biosystems, Foster City, CA, USA) and by spectrophotometer (U-2000, Hitachi, Tokyo, Japan) with a wavelength set at 520 mm respectively. Means and standard deviations (SD) of urinary cotinine levels with creatinine correction were 1.93±3.74 mg/g in 22 smokers and 0.0032±0.0054 mg/g in 74 non-smokers (*p*<0.0001) [27].

For verifying alcohol drinking, we measured serum acetaldehyde, a carcinogen in animals and major metabolite of ethanol, in 35 non-smoking drinkers and 19 non-smoking non-drinkers, all healthy, by the technique of a high-performance liquid chromatography (HPLC, Beckman Coulter Module 126, UK) [28], [29]. Means and SDs were 1481.78±699.37 ppb in 35 non-smoking drinkers and 996.48±247.22 ppb in 19 non-smoking non-drinkers (*p* = 0.0012). Since areca nuts in Taiwan contain a high concentration of safrole, a carcinogen, our previous study has analyzed safrole-DNA adducts using the P-postlabelling method in 47 tissue specimens of esophageal cancer (16 areca chewers and 31 non-areca chewers) [30]. Safrole-DNA adducts were detected in 5 (31.3%) out of 16 areca chewers and, in contrast, detected in none of the 31 non-areca chewers (Fischer's exact test, *p* = 0.0028) [31].

### Statistic Analysis

Means ± SD and medians (interquartile range, IQR) of ages were compared according to the category of demographic variables and clinical stage. Differences of means between two or three groups were tested using the independent *t*-test or ANOVA statistics.

The main interest of this study was to investigate the relationship of substances uses (alcohol, tobacco, and areca nut) with age-onset of ESCC. In addition to the effect of ever-use of these substances, we examined whether age at start and daily average, if uses, of consuming these substance affected the age-onset of ESCC. For the duration (years) of substance uses, we found the high correlation between age of study subjects and years of alcohol drinking, cigarette smoking, and areca nut chewing (Spearman correlation co-efficiencies r = 0.64, 0.66, and 0.44, respectively, all *p*-values<0.0001). Thus the variables of duration of substance uses were not examined.

Multiple linear regressions were used to explore the relationship of age at the diagnosis of ESCC with different substance uses, which were significant in the univariate analysis. Although the variable of clinical stage did not associate with the diagnosed age of ESCC, we still placed it in the multivariate analyses. All tests were performed by SAS 9.1 statistical software; two-sided *p* value<0.05 was considered as significant.

## Results

In total, 668 case patients, all male, were analyzed. The mean age (±SD) at presentation of ESCC was 59.2 (±11.3) years. Subjects with educational level of high school were diagnosed ESCC younger than the other two groups (Table S1). Regarding the distribution of clinical stage, 63 (9.4%) were stage I, 169 (25.3%) were stage II, 336 (50.3%) were stage III, and 100 (15.0%) were stage IV of ESCC. Mean ages of initial presentation were comparable in all stage groups (*p* = 0.65). Since the mean age of subjects diagnosed in KMUH was approximately 4 years younger than those in the other two hospitals, we analyzed patient number and frequency in different clinical stages by KMUH (n = 158) and non-KMUH (n = 510). We found that patient numbers (%) of clinical stage I, II, III and IV were 7 (4.4%), 37 (23.4%), 68 (43.0%), and 46 (23.1%) in KMUH and 56 (11.0%), 132 (25.9%), 268 (52.5%), and 54 (10.6%) in non-KMUH respectively. Although it was significantly different (χ^2^ = 35.60; d.f. = 3; *p*<0.0001), KMUH had less subjects with stage I and more subjects with stage IV than non-KMUH.

Of the 668 cases, 543 (81.3%) reported habitual alcohol drinking, 580 (86.8%) cigarette smoking, and 298 (44.6%) areca nut chewing. All substance users, including alcohol drinkers, cigarette smokers, and areca nut chewers, were, on average, diagnosed with ESCC younger than non-drinkers, non-smokers, and non-chewers (Table 1). After adjusting for educational levels, study hospitals, clinical stages, cigarette smoking, and areca nut chewing, we found that alcohol drinkers had ESCC 3.58 years younger than non-drinkers (*p* = 0.002). A similar result was found in areca nut chewers, who had ESCC 6.34 years younger than non-chewers (*p*<0.0001), but not in cigarette smokers (*p* = 0.10) (Table 1).

**Table 1:** Relationship of substance uses with diagnosed age of esophageal squamous cell carcinoma.

Variables	Number	Mean ± SD	Median (IQR)	*P*-value	β (95% CI)^a^	*P*-value^a^
Overall	668	59.2±11.3	59 (67, 50)			
Alcohol drinking						
No	125	63.9±11.8	65 (55, 73)	<0.0001	-	0.002
Yes	543	58.1±10.9	58 (50, 66)		−3.58 (−5.86, −1.30)^a^	
Age at starting drinking						
≥20 yrs	430	58.7±10.8	59 (50, 66)	0.005	-	0.07
<20 yrs	113	55.9±11.1	55 (48, 63)		−1.39 (−2.90, 0.12)^b^	
Daily average						
<3 drinks/day	144	59.9±11.2	60 (52, 68)	0.02	-	0.02
≥3 drinks/day	399	57.4±10.7	57 (49, 65)		−2.54 (−0.48, −4.61)^c^	
Cigarette smoking						
No	88	61.4±11.5	60 (53, 70)	0.05	-	0.10
Yes	580	58.8±11.2	59 (50, 67)		2.20 (−0.42, 4.82)^a^	
Age at starting smoking						
≥20 yrs	356	61.2±10.7	62 (53, 68)	<0.0001	-	0.13
<20 yrs	224	55.0±11.0	54 (47, 62)		−1.08 (−2.46, 0.31)^b^	
Daily average						
<20 cigarettes/day	420	57.5±10.0	57 (50, 64)	0.07	-	0.11
≥20 cigarettes/day	160	59.3±11.7	60 (50, 68)		−1.64 (−3.68, 0.40)^c^	
Areca chewing				<0.0001		
No	370	62.3±11.8	64 (54, 71)		-	<0.0001
Yes	298	55.3±9.2	55 (48, 62)		−6.34 (−7.98, −4.70)^a^	
Age at starting chewing						
≥20 yrs	235	56.2±8.9	56 (49, 63)	0.0002	-	<0.0001
<20 yrs	63	51.5±9.5	51 (44, 58)		−4.51 (−3.23, −5.78)^b^	
Daily average						
<20 betel nuts/day	213	55.2±9.5	54 (48, 61)	0.79	-	0.78
≥20 betel nuts/day	85	55.5±8.6	56 (49, 62)		0.34 (−2.03, 2.71)^c^	

Abbreviations: IQR: Interquartile range; CI: Confidence interval; KMUH: Kaohsiung Medical University Hospital; KVGH: Kaohsiung Veterans General Hospital; NTUH: National Taiwan University Hospital.

a Adjusting for educational levels (>high school *vs.* <high school; high school *vs.* <high school), study hospitals (KMUH *vs.* NTUH; KVGH *vs.* NTUH), clinical stage, and substance uses (tobacco, alcohol, or areca: yes *vs.* no).

b Compared to ≥20 year of age starting to smoking, drinking or chewing.

c Compared to <3 drinks/day in alcohol drinking, <20 cigarettes/day in cigarette smoking, or <20 betel nuts/day in areca chewing.

Among subjects with alcohol drinking (n = 543), we found that subjects consumed, on average, ≥3 drinks per day had younger age to have ESCC than those consumed <3 drinks per day (*p* = 0.02). The same significant result was not found in age at starting drinking (<20 yrs *vs.* ≥20 yrs). In contrast, among subjects with areca nut use (n = 298), we found that subjects who started chewing before age 20 had, on average, ESCC at a significantly younger age than those who started at an older age (≥20 years of age) (*p*<0.0001). Similar results were not found in alcohol drinkers (*p* = 0.07) or cigarette smokers (*p* = 0.13). The same significant result was not found in daily average of areca nut chewing (≥20 betel nuts/day *vs.* <20 betel nuts/day). Both age at start of smoking and daily average of smoking were not significantly associated with age-onset of ESCC (Table 1).

Our previous case-controlled study of 513 ESCC case patients and 818 gender- and age-matched controls had demonstrated that users of any two of the three substances can contract at least a 4-fold risk of ESCC [12]. This risk increased up to 41-fold for users of all three substances. Thus, using the combined non- and single-substance users as a comparison group and adjusting for educational levels, study hospitals, and clinical stages, subjects with the habits of both cigarette smoking and alcohol drinking were nearly 3 years younger to have ESCC (Table 2). Users of areca plus another substance had even younger mean ages (7∼8 years younger) than the comparison group. Subjects who use all three substances had the largest age difference, being 9.20 years younger than the comparison group (*p*<0.0001). The results remained consistent after categorized by clinical stage (early stage, stage I and II *vs.* late stage, stage III and IV) (Table 3) or study hospitals (NTUH in Taipei *vs.* KMUH and KVGH in Kaohsiung) (Table S2).

**Table 2:** Association between number of substance uses and diagnosed age of esophageal squamous cell carcinoma.

Alcohol	Tobacco	Areca	n	Mean ± SD	Median (IQR)	β (95% CI)	*p*-value	Adjusted β^a^ (95% CI)	*p*-value
≤1 substance use			121	65.2±11.5	67 (56, 75)	-	-	-	-
+	+	−	249	60.9±11.7	63 (52, 69)	−4.29 (−1.97, −6.61)	<0.0001	−3.45 (−1.25, −5.66)	0.002
+	−	+	11	54.5±8.6	57 (48, 60)	−10.65 (−4.06, −17.25)	0.002	−8.07 (−1.80, −14.33)	0.01
−	+	+	23	56.6±9.9	55 (48, 63)	−8.59 (−3.83, −13.35)	<0.0001	−7.78 (−3.21, −12.34)	0.001
+	+	+	264	55.2±9.2	55 (48, 61)	−10.03 (−7.73, −12.33)	<0.0001	−9.20 (−7.00, −11.39)	<0.0001

Abbreviations: SD, standard deviation; IQR, interquartile range; CI, confidence interval.

a Adjusting for education levels, study hospitals and clinical stage.

**Table 3:** Association between number of substances used and the diagnosing age of esophageal squamous cell carcinoma categorized by clinical stages.

Alcohol	Tobacco	Areca	n	Mean ± SD	Median (IQR)	β (95% CI)	*p*-value	Adjusted β^a^ (95% CI)	*p*-value
Clinical stageEarly stage (I and II, n = 232)									
≤1 substance use			51	65.0±10.8	66 (56, 75)	-	-	-	-
+	+	−	86	60.6±11.0	62 (52, 68)	−4.40 (−8.02, −0.79)	0.02	−3.28 (−6.84, 0.29)	0.07
+	−	+	3	57.0±11.5	53 (48, 70)	−8.02 (−20.18, 4.14)	0.20	−8.16 (−19.87, 3.55)	0.17
−	+	+	9	57.1±10.8	63 (48, 65)	−7.91 (−15.31, −0.51)	0.04	−7.44 (−14.76, −0.12)	0.05
+	+	+	83	56.4±9.3	55 (50, 63)	−8.63 (−12.28, −4.99)	<0.0001	−7.65 (−11.21, −4.09)	<0.0001
Late stage (III and IV, n = 436)									
≤1 substance use			70	65.3±12.1	68 (56, 74)	-	-	-	-
+	+	−	163	61.1±12.1	63 (52, 69)	−4.27 (−7.31, −1.22)	0.006	−3.95 (−6.79, −1.12)	0.006
+	−	+	8	53.6±8.0	57 (48, 60)	−11.70 (−19.66, −3.75)	0.004	−7.85 (−15.31, −0.38)	0.04
−	+	+	14	56.3±9.8	55 (48, 63)	−9.04 (−15.29, −2.80)	0.005	−8.43 (−14.28, −2.58)	0.005
+	+	+	181	54.6±9.2	55 (48, 61)	−10.72 (−13.72, −7.72)	<0.0001	−10.19 (−13.00, −7.38)	<0.0001

Abbreviations: SD, standard deviation; IQR, interquartile range; CI, confidence interval.

a Adjusting for education levels and study hospitals (NTUH *vs.* KMUH and KVGH).

## Discussion

In this study we have found that, among the substances most linked to ESCC, alcohol and areca nut are associated with earlier development of the disease. However, similar result was not observed with tobacco use alone. It is well established that long-term cigarette smoking is associated with various cardiovascular and pulmonary diseases and cancers resulting in reduced life-expectancy in long-term smokers [32]. Since the premature death rate is markedly higher among smokers [33], we speculate that these competing risks may cause some smokers to die before cancer of the esophagus was present thereby affecting results in the mean presenting age of ESCC.

Subjects who drank, on average, alcohol beverages ≥3 drinks per day were diagnosed with ESCC approximately 2.5 years younger than those <3 drinks per day, whereas subjects starting to chew areca nut before age 20 were diagnosed with ESCC approximately 4.5 years younger than those starting after age 20. In addition, areca nut use in combination with alcohol or cigarette or both was associated with at least 4 years earlier of the diagnosis of ESCC, when compared to use of cigarette and alcohol only (Table 2). Our previous epidemiological study of 513 ESCC case patients and 818 gender- and age-matched controls found that users of areca plus another substance (alcohol or tobacco) had a 4–14-fold increase in the risk of ESCC [12], but none of these 513 case patients used areca only. In contrast, 5 out of 818 healthy controls used only areca [12]. These results may suggest that areca plays a role in promoting the carcinogenesis of the esophagus. Indeed, *in vitro* studies have suggested that arecoline, one major alkaloid of areca nut, could promote genomic instability through arresting cells at prometaphase with large amounts of misaligned chromosomes and accelerate keratinocyte inflammation by regulating cytokines production such as interleukin-6 and TNF-alpha [30], [34].

The mean age at diagnosis of ESCC in the group of 0–1 substance use was 65 years, very similar to the report (67 years) from the cancer statistical data of the US National Cancer Institute [23]. In addition, previous observations have found more than half of ESCC patients had advanced cancer at presentation [4]. In our study 65.3% (436 out of the 668) of ESCC patients were diagnosed at late stages (stages III or IV). It has been established that the initial staging greatly affects the survival rate of patients with ESCC [5], [35]. Given relatively high prevalence of ESCC in Taiwan and its' tendency to present late in the disease course, a strategy of periodic screening for high-risk populations may be warranted.

The appropriate age to start screening for ESCC in high-risk groups has not yet been established, although one study has screened residents over age 35 using esophageal balloon cytology in Anyang County of China, where the incidence of esophageal cancer was high [36]. This study surveyed 20,049 persons and 1,018 had grade 2 dysplasia or worse, 164 of which had invasive cancer. Since 2000, the American Cancer Society has annually published cancer screening guidelines which include cancers of the breast, cervix, colorectum, endometrium, lung, prostate, and the skin [23]. The recommendations have not covered esophageal cancer. Main reasons may be the lack of optimal non-invasive screening tool for esophageal cancer, and the inconclusive findings on the definition of high-risk group for esophageal cancer, especially for ESCC. Since the combined effects of tobacco, alcohol, and areca use account for 83.7% of the attributable fraction of contracting ESCC [12], [19], [37], people in this high-risk group are highly recommended to have their aero-digestive system closely monitored including the oral cavity, pharynx, larynx, and esophagus [10], [12], [17], [37].

The appropriate interval at which high risk, cancer-free men should repeat screening for ESCC is unclear, although a screening of patients with Barrett's esophagus without epithelial dysplasia has been recommended using endoscopy every 3–5 years [24]. Wang and colleagues conducted an 11-year follow-up of 578 subjects from Anyang County and nearby Linxian of Henan Province, an area recognized as having the highest incidence of ESCC in China. Of the initially cancer-free subjects, 23 developed early stage esophageal cancer during follow-up. The mean time of cancer development (from entry of the study to cancer detection) was 5.0±2.9 years in males and 4.7±3.2 years in females; however, the interval at which these subjects received endoscopic examination was not reported [38]. Thus, the appropriate frequency of screening the digestive system for cancer (especially ESCC) is yet to be determined, and further investigation is needed.

The exposure of interest in our study was measured by questionnaires, and recall bias is likely. The accuracy of information on substance use in this study has been verified by different biomarkers from different specimens to reduce the possibility of information bias. Table 3 and Table S2 shows that the correlation between habitual substance use and the age of ESCC diagnosis is consistent, even after being dichotomized by clinical stage (early stage *vs.* late stage) or study hospital (NTUH in Taipei *vs.* KMUH and KVGH in Kaohsiung), suggesting the findings were not confounded by other covariates. The mean age of subjects diagnosed in KMUH was approximately 4 years younger than those in the other two hospitals; however, KMUH had less subjects with early stage (stage I) and more subjects with advanced stage (stage IV) than non-KMUH. These results suggest younger people would visit the gastroenterologists for help, when their ESCC-related symptoms were already significant. Although educational levels may not entirely represent for socioeconomic status, given the question about the revenue or salary in the study family in Taiwan is a sensitive one, the data about socioeconomic status from interviewing study patients can be likely introduced the bias.

This is a case-control study design. Although we found that subjects who used two to three substances, daily drank alcohol beverages ≥3 drinks, or started to chew areca before 20 years of age had the tendency of a younger age to develop ESCC, these observations are necessary to be reconfirmed in the large-scale prospective cohort studies to recommend the screening program of an early ESCC in the high-risk people.

## Supporting Information

Figure S1 **Secular incidence and mortality rate by gender (A: Male, B: Female) in Taiwan, 1995–2007.**
(TIF)

Table S1 **Relationship of demographic and clinical characteristics with diagnosed age of esophageal squamous cell carcinoma.**
(DOC)

Table S2 **Association between number of substances used and the diagnosing age of esophageal squamous cell carcinoma categorized by study hospitals.**
(DOC)
