# Supplementary figures and images for: Areca Users in Combination with Tobacco and Alcohol Use Are Associated with Younger Age of Diagnosed Esophageal Cancer in Taiwanese Men
Source: PLoS One. 2011 Oct 19;6(10):e25347. doi: 10.1371/journal.pone.0025347 (PMC3198438; doi:10.1371/journal.pone.0025347)

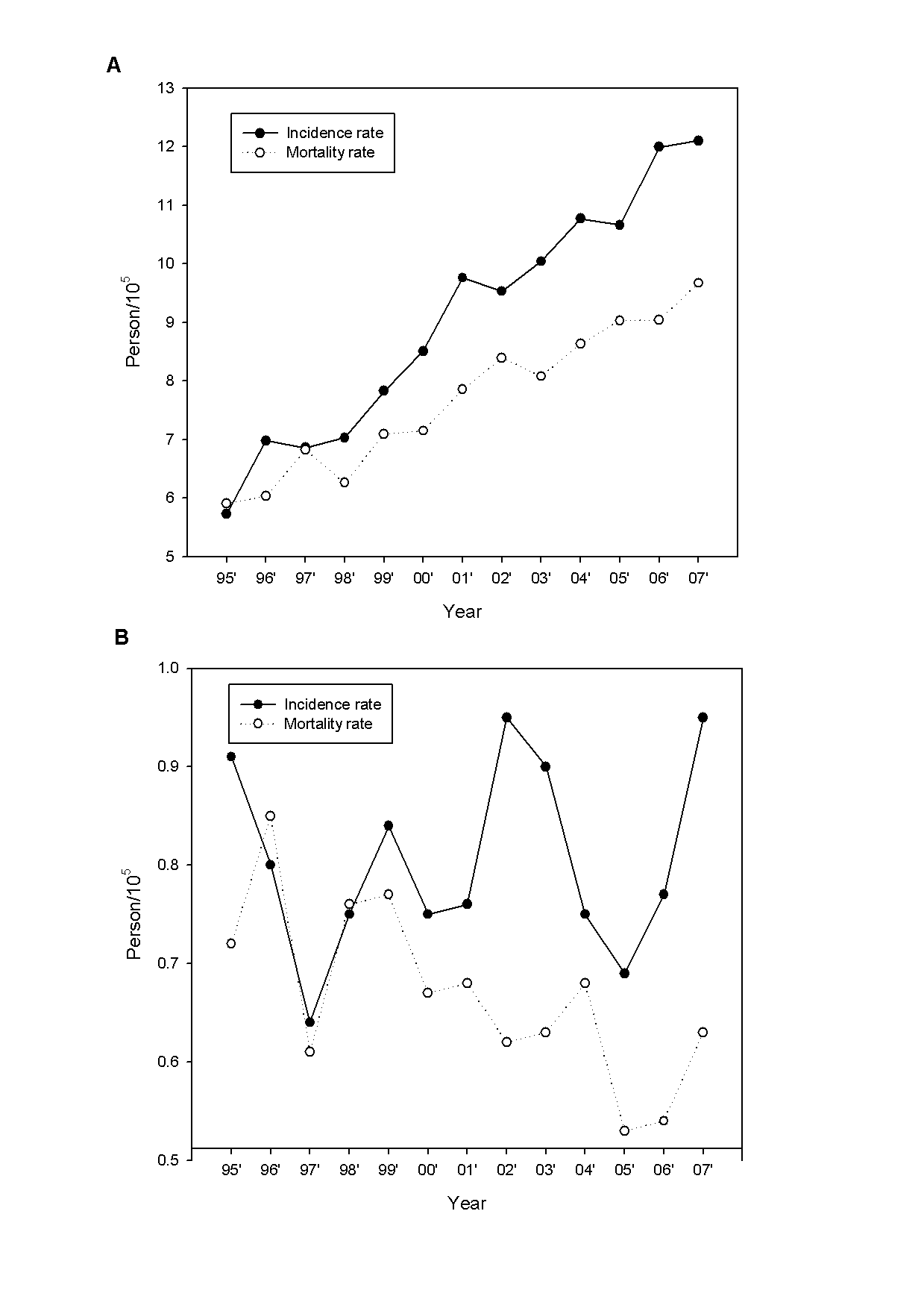

Supplement: Figure S1 — Secular incidence and mortality rate by gender (A: Male, B: Female) in Taiwan, 1995–2007. (TIF) [file pone.0025347.s001.tif]
