# Supplementary material for: Areca Users in Combination with Tobacco and Alcohol Use Are Associated with Younger Age of Diagnosed Esophageal Cancer in Taiwanese Men
Source: PLoS One. 2011 Oct 19;6(10):e25347. doi: 10.1371/journal.pone.0025347 (PMC3198438; doi:10.1371/journal.pone.0025347)
Supplement: Table S1 — Relationship of demographic and clinical characteristics with diagnosed age of esophageal squamous cell carcinoma. (DOC) [file pone.0025347.s002.doc]

**Table S1. Relationship of demographic and clinical characteristics with diagnosed age of esophageal squamous cell carcinoma.**

| **Variables** | **Number** | **Mean ± SD** | **Median (IQR)** | ***P*-value** |
| --- | --- | --- | --- | --- |
| Overall | 668 | 59.2 ±11.3 | 59 (67, 50) |  |
| Educational levels |  |  |  |  |
| < High school | 358 | 62.6 ± 9.8 | 63 (55, 70) | < 0.0001 |
| High school | 245 | 54.4 ± 11.0 | 53 (46, 61) |
| > High school | 65 | 58.3 ± 13.3 | 57 (48, 67) |
| Study Hospitals |  |  |  |  |
| KMUH | 158 | 56.2 ± 10.8 | 57 (48, 65) | 0.0008 |
| KVGH | 167 | 60.0 ± 12.0 | 60 (50, 70) |
| NTUH | 343 | 60.1 ± 11.0 | 60 (51, 68) |
| Clinical stage |  |  |  |  |
| Stage 1 | 63 | 60.4 ± 10.8 | 60 (52, 70) | 0.65 |
| Stage 2 | 169 | 59.7 ± 10.8 | 60 (52, 70) |  |
| Stage 3 | 336 | 58.8 ± 11.5 | 59 (50, 67) |  |
| Stage 4 | 100 | 58.7 ± 11.7 | 57 (49, 67) |  |
