# Supplementary material for: Areca Users in Combination with Tobacco and Alcohol Use Are Associated with Younger Age of Diagnosed Esophageal Cancer in Taiwanese Men
Source: PLoS One. 2011 Oct 19;6(10):e25347. doi: 10.1371/journal.pone.0025347 (PMC3198438; doi:10.1371/journal.pone.0025347)
Supplement: Table S2 — Association between number of substances used and the diagnosing age of esophageal squamous cell carcinoma categorized by study hospitals. (DOC) [file pone.0025347.s003.doc]

**Table S2. Association between number of substances used and the diagnosing age of esophageal squamous cell carcinoma categorized by study hospitals.**

| **Alcohol** | **Tobacco** | **Areca** | **n** | **Mean ± SD** | | **Median (IQR)** | | **β (95% CI)** | | ***p*-value** | | **Adjusted β^a^ (95% CI)** | | ***p*-value** | | | |  |
| --- | --- | --- | --- | --- | --- | --- | --- | --- | --- | --- | --- | --- | --- | --- | --- | --- | --- | --- |
| Study hospitals  NTUH in Taipei (n=343) | | | | |  |  |  | |  | |  | |  | |  | |  | |
| ≤ 1 substance use | | | 74 | 64.6 ± 11.2 | | 66 (56, 73) | | - | | - | | - | | - | | | |  |
| + | + | - | 141 | 61.1 ± 11.1 | | 62 (53, 69) | | -3.47 (-6.44, -0.50) | | 0.02 | | -2.96 (-5.87, -0.06) | | 0.05 | | | |  |
| + | - | + | 5 | 50.4 ± 7.0 | | 50 (45, 57) | | -14.17 (-23.74, -4.60) | | 0.004 | | -11.15 (-20.43, -1.88) | | 0.02 | | | |  |
| - | + | + | 4 | 52.5 ± 12.8 | | 53 (41, 64) | | -12.07 (-22.70, -1.44) | | 0.03 | | -10.70 (-20.93, -0.48) | | 0.04 | | | |  |
| + | + | + | 119 | 56.8 ± 9.4 | | 55 (49, 64) | | -7.74 (-10.81, -4.68) | | <0.0001 | | -7.10 (-10.05, -4.14) | | <0.0001 | | | |  |
| KMUH & KVGH in  Kaohsiung (n=325) | | | | |  |  |  | |  | |  | |  | | |  | | |
| ≤ 1 substance use | | | 47 | 66.2 ± 12.0 | | 71 (56, 75) | | - | | - | | - | | - | | | |  |
| + | + | - | 108 | 60.7 ± 12.2 | | 64 (51, 70) | | -5.53 (-9.23, -1.84) | | 0.003 | | -4.43 (-7.91, -0.96) | | 0.01 | | | |  |
| + | - | + | 6 | 58.0 ± 8.8 | | 58 (54, 64) | | -8.19 (-17.37, 0.98) | | 0.08 | | -5.67 (-14.34, 2.99) | | 0.20 | | | |  |
| - | + | + | 19 | 57.5 ± 9.4 | | 55 (50, 63) | | -8.72 (-14.47, -2.96) | | 0.003 | | -7.62 (-13.03, -2.20) | | 0.006 | | | |  |
| + | + | + | 145 | 53.8 ± 8.9 | | 53 (48, 61) | | -12.39 (-15.94, -8.83) | | <0.0001 | | -11.48 (-14.83, -8.13) | | <0.0001 | | | |  |

Abbreviations: SD, standard deviation; IQR, interquartile range; CI, confidence interval; NTUH: National Taiwan University Hospital; KMUH: Kaohsiung Medical University Hospital; KVGH: Kaohsiung Veteran General Hospital.

^a^Adjusting for education levels and clinical stages (early stage *vs.* late stage).
